# Flavocytochrome ***b*_2_**-Based Enzymatic Method of L-Lactate Assay in Food Products

**DOI:** 10.1155/2013/461284

**Published:** 2013-09-24

**Authors:** Oleh Smutok, Maria Karkovska, Halyna Smutok, Mykhailo Gonchar

**Affiliations:** ^1^Department of Analytical Biothecnology, Institute of Cell Biology, Drahomanov Street 14/16, Lviv 79005, Ukraine; ^2^Institute of Applied Biotechnology and Basic Sciences, University of Rzeszow, Sokolowska Street 26, 36-100 Kolbuszowa, Poland

## Abstract

L-lactate, a key metabolite of the anaerobic glycolytic pathway, plays an important role as a biomarker in medicine, in the nutritional sector and food quality control. For these reasons, there is a need for very specific, sensitive, and simple analytical methods for the accurate L-lactate measuring. A new highly selective enzymatic method for L-lactate determination based on the use of flavocytochrome *b*_2_ (EC 1.1.2.3; FC *b*_2_) isolated from the recombinant strain of the yeast *Hansenula polymorpha* has been developed. A proposed enzymatic method exploits an enzymatic oxidation of L-lactate to pyruvate coupled with nitrotetrazolium blue (NTZB) reduction to a colored product, formazan. The maximal absorption peak of the colored product is near *λ* = 525 nm and the linear range is observed in the interval 0.005–0.14 mM of L-lactate. The main advantages of the proposed method when compared to the LDH-based routine approaches are a higher sensitivity (2.0 **μ**M of L-lactate), simple procedure of analysis, usage of inexpensive, nontoxic reagents, and small amount of the enzyme. Enzymatic oxidation of L-lactate catalyzed by flavocytochrome *b*_2_ and coupled with formazan production from nitrotetrazolium blue was shown to be used for L-lactate assay in food samples. A high correlation between results of the proposed method and reference ones proves the possibility to use flavocytochrome *b*_2_-catalysed reaction for enzymatic measurement of L-lactate in biotechnology and food chemistry.

## 1. Introduction

Lactic acid is a universal metabolite of nearly all living organisms and a natural or artificial component of many food products. An accurate quantitative determination of L-lactate is very important for fermentation industries, online control of the quality of alcoholic beverages [1], and sour milk products [2]. Moreover, detection of L-lactate is important for control of the quality of food which contain lactate salts or free acid as pH-stabilizing additive and preservative (especially in meat's products for growth reducing the some pathogenic bacteria like *Listeria monocytogenes*) [3]. The increase of lactate concentration in some technological processes can be a result of microbial contamination [4]; therefore, a precise lactate assay is necessary for detection of early stages of such processes. In addition, many cosmetics, pharmaceuticals, domestic washing powders, and antiseptic goods contain this compound [5]. A level of L-lactate content in human blood is an important clinical indicator of hypoxia, acidosis [6], and level of drug's toxicity [7] and serves as a marker for evaluation of the optimal sportsmen's training [8]. L-lactate is also an important biomarker for different types of cancer due to Warburg phenomenon [9].

For an assay of L-lactate, a lot of physicochemical and chemical methods have been proposed: spectrophotometry [10], fluorometry [11], pH potentiometric measurements [12], and amperometric biosensors based on O_2_ and H_2_O_2_ electrodes [13]. Most of these methods require a lot of time and previous labor-consuming procedures such as filtration, chromatography, and deproteinization. On the other hand, most of them require expensive equipment or are non-selective.

Enzyme-based spectrophotometric assay of L-lactate is widely used due to the favorable sensitivity of biorecognizing compounds and simple procedure of analysis. Among available methods for the determination of L-lactate by enzymatic spectrophotometric approaches, the most exploited are the methods with the use of NAD^+^-dependent lactate dehydrogenase (LDH) from animal muscles or heart (EC 1.1.1.27) [14] or bacterial lactate oxidase (LOX) (EC 1.13.12.4) [15]. Weak points of LDH-based approaches are high price of the enzyme and coenzyme and nonsufficient selectivity. Moreover, NAD^+^-dependent LDH equilibrium is not optimal for L-Lactate assay, so the reaction of the product (pyruvate) with toxic compounds, for example, hydrazine or hydroxylamine, or the use of an additional enzyme such as glutamate-pyruvate transaminase (GPT) is necessary to shift the equilibrium to the product side [16]. On the other hand, elimination of reverse effects could be performed by higher pH value and NAD^+^content [17]. The imperfections of LOX-based methods of L-lactate analysis are their expensiveness and interference at a high protein content in the analyzed samples [15].

Besides LDL and LO, another enzyme is known for participating in the lactic acid metabolism in yeast, namely, L-lactate-cytochrome *c* oxidoreductase (EC 1.1.2.3; flavocytochrome *b*_2_, FC *b*_2_) [18] which catalyses the electron transfer from L-lactate to cytochrome *c* in yeast mitochondria. The protein from *Saccharomyces cerevisiae *and *Hansenula anomala *is a tetramer with four identical subunits, each consisting of FMN- and heme-binding domains [19]. FC *b*_2_ has absolute specificity for L-lactate; moreover, it functions *in vitro* with no regard to the nature of electron acceptors which makes this enzyme very promising for analytical biotechnology.

In this paper, we describe a new enzymatic method for L-lactate assay based on the use of a purified FC *b*_2_, isolated from the recombinant strain of the thermotolerant yeast *Hansenula polymorpha*, coupled with phenazine-mediated reduction of nitrotetrazolium salt to photometrically detected colored formazan product. Recently, we have reported about the development of a highly selective method/kit for L-lactate analysis based on FC *b*_2_-dependent enzymatic transformation of L-lactate coupled with nonenzymatic generation of dissolved form of Prussian Blue at the presence of specific solubilizer [20, 21]. Moreover, possibility of FC *b*_2_ using as L-lactate selective element in biosensor technology has been successfully proved by us previously [22, 23]. 

The aim of the work is using flavocytochrome *b*_2_-catalyzed reaction for L-lactate assay in food products. The peculiarity of the proposed bioanalytic method is its high selectivity, usage of inexpensive and nontoxic reagents that provide a simple, accurate assay procedure and a low price of the analysis. It is worth to mention that contrary to a very unstable *S*. *cerevisiae*-derived enzyme, FC *b*_2_ for thermotolerant species *H. polymorpha *is stable and better fits the analytical purposes.

## 2. Material and Methods

### 2.1. Materials 

DEAE-Toyopearl 650M was obtained from Toyo Soda (Tokyo, Japan). EDTA and nitrotetrazolium blue (NTZB) were from Merck (Darmstadt, Germany). L(+)-Lactic acid was from Acros Organics (Geel, Belgium). Sodium L-lactate and LDH from bovine heart (EC 1.1.1.27) were purchased from Sigma-Aldrich Corporation (Deisenhofen, Germany), Triton X-100 from Fluka (Buchs, Switzerland), and NAD^+^ and NADH were obtained from Gerbu Biotechnik (Gailberg, Germany), LOX-based sensor from SensLab GmbH (Leipzig, Germany). All chemicals and reagents were of analytical grade and all solutions were prepared using HPLC-grade water. L-lactate standard solution and appropriate dilutions were prepared in 100 mM phosphate buffer, pH 7.8.

### 2.2. Isolation and Purification of L-Lactate Cytochrome *c* Oxidoreductase (FC *b*_2_)

The recombinant strain tr1 of the thermotolerant yeast *Hansenula polymorpha *possessing enzyme with a specific activity up to 11 U·mg^−1^ in cell-free extracts [29] was used as a source of FC *b*_2_. Enzyme was isolated by ion-exchange chromatography on DEAE-Toyopearl cellulose 650 M [18]. The purified enzyme was stored as a suspension in 70%-saturated ammonium sulfate at −10°C before using.

### 2.3. Assay of FC *b*_2_ Activity

One unit of the FC *b*_2_ activity is defined as that amount of the enzyme which forms 1 *μ*mol hexacyanoferrate(II) per minute under standard conditions of the assay (20°C, 30 mM phosphate buffer, pH 7.8). Activity was estimated by spectrophotometric monitoring of hexacyanofer rate(III) reduction at *λ* = 420 nm. During this process, optical density of the analyzed solution becomes lower. Assay mixture consisted of 30 mM phosphate buffer, pH 8.0, 33 mM sodium L-lactate, 1 mM EDTA, and 83 mM K_3_Fe(CN)_6_.

The specific activity of FC *b*_2_ was calculated by formula:

(1) $$\begin{array}{c} \text{SA}=\frac{\Delta E/\min\cdot V\cdot n}{{ℰ}_{\text{mM}}\cdot Cp\cdot V_{E}}, \end{array}$$

where Δ*E*/min⁡—change of optical density at *λ* = 420 nm per min; *V*—total volume of the assay solution, mL; *n*—dilution of the enzyme before assay; *ℰ*_mM_—millimolar extinction of hexacyanoferrate(III), 1.04 mM^−1^·cm^−1^; *V*_*E*_—volume of the added enzyme aliquot, mL; *Cp*—protein concentration of the tested FC *b*_2_ solution determined by the Lowry method, mg·mL^−1^.

### 2.4. Enzymatic Assay of L-Lactate

Optimized reaction mixture consisted of 50 mM phosphate buffer, pH 7.8; 1.5 mM phenazine methosulfate; 0.1% nitrotetrazolium blue; 1% Triton X-100; 0.5 U·mL^−1^ FC *b*_2_. The assay was carried out in the dark at 25°C. The reaction was started by adding 0.2 mL analyzed sample into a glass test tube with 0.8 mL reaction mixture. Photometric measurements were carried out on spectrophotometer *SHIMADZU UV-1650 PC* at 525 nm in plastic cuvette after incubation of the samples during 20 min in the dark. The blank sample consisted of all reagents, except lactate (water was added instead of analyzed sample). The reaction was terminated by adding 3 mL of 0.3 M HCl. The calibration was carried out using standard solution of L-lactate (60 mM) in 40-fold dilutions.

### 2.5. Preparation of Food Products and Wines

The milk samples were mixed with 4% trichloroacetic acid (final concentration). The supernatants were neutralized by 1 M KOH and used for analysis. The ketchup samples were diluted with distilled water and filtrated through 0.2 *μ*m pores microfilter Minisart NML SM 16534 K (Sartorius GmbH, Gottingen, Germany). The wine samples were diluted for 50 times by distilled water. Additional treatment of the wine and juices samples was omitted.

## 3. Results and Discussion

### 3.1. Development of an Enzymatic L-Lactate Assay

**Figure 1:**
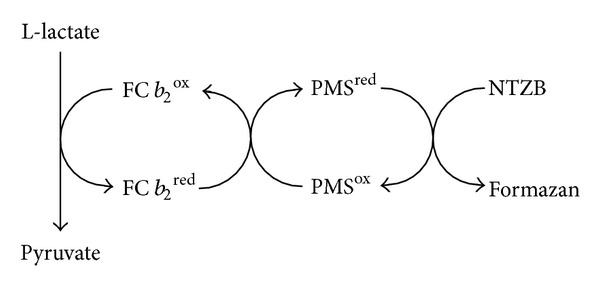
The scheme of the reactions exploited in enzymatic assay of L-lactate.

The procedure is based on FC *b*_2_-catalyzed oxidation of L-lactate to pyruvate. Different mediators (hexacyanoferrate(III), methylene blue, phenazine ethosulfate, and phenazine methosulfate) were tested to provide an optimal electron transfer from the reduced enzyme to nitrotetrazolium blue (NTZB). Phenazine methosulfate (PMS) was chosen as the best mediator (data not shown). To provide solubilization of the formed formazan, a detergent Triton X-100 in 1% final concentration was used. The principle of the proposed method is shown in Figure 1.

The optimal conditions for NTZB reduction to colored product formazan were investigated. An optimal time of incubation duration at temperature 20–25°C is about 20 min for the activity of FC *b*_2_ in reaction mixture 0.5 U·mL^−1^. The maximal absorption of the reduction product was observed at 525 nm at acidic conditions.

**Figure 2:**
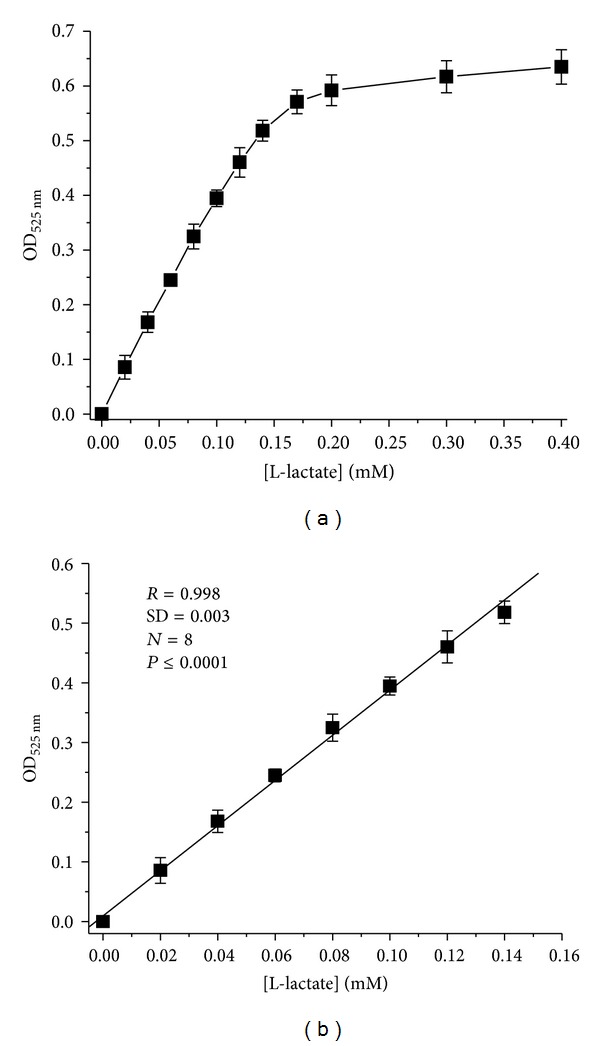
Dependence of optical density of the reaction mixture on L-lactate concentration (a) and a linear range (b) for enzymatic method. Conditions are described in Section 2.

As shown in Figure 2, formazan formation is linearly dependent on the concentration of L-lactate in the reaction mixture up to 0.14 mM. In the indicated concentration range, the formazan product which has usually a low solubility was not precipitated (in the presence of Triton X-100). Optical density of the reaction mixture after adding HCl was stable for several days (in the dark). 

The proposed method has the next operational parameters: limit of assay—about 2 *μ*M of L-lactate (in final photometred mixture); linear range—up to 0.14 mM of L-lactate.

The analytical parameters of the proposed enzymatic method in comparison with the known approaches are presented in Table 1.

**Figure 3:**
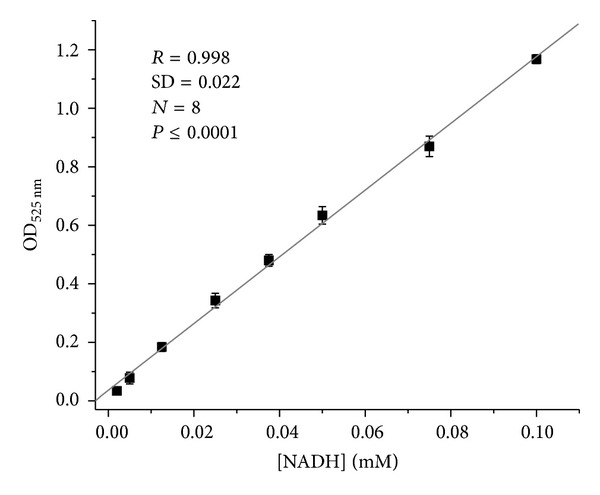
Dependence of optical density of reaction mixture on NADH concentration.

To estimate the level of the analyte transformation to the final product, additional calibration using NADH as a reducing agent for NTZB was carried out. For this procedure, conditions were the same as for L-lactate assay, except for the addition of FC *b*_2_. The precise concentration of NADH standard solution was calculated by measuring its optical density at 340 nm (*ℰ*_mM_ = 6.23 mM^−1^·cm^−1^). Calibration curve for NADH in PMS-NTZB-formazan reaction is shown in Figure 3.

On the base of molar extinction of formazan product calculated from calibration data obtained in reaction NADH + PMS + NTZB, the enzymatic conversion of the analyte under the used conditions was about 40% that is in a good correlation with units of FC *b*_2_ activity added to reaction mixture.

### 3.2. Assay of L-Lactate in Some of Food Products and Wine

The developed method was tested on samples of food products(milk, ketchup, and juice). The results obtained by this approach have been compared with the reference methods: biosensor analysis using SensLab biosensor [27], NAD^+^-dependent LDH spectrophotometric method [28], and Prussian Blue generation [20, 21] (Table 2).

Generally, the obtained results of L-lactate analysis in food products are in a good correlation with the compared methods that confirms the correctness of lactate assay by the developed approach. 

The content of L-lactate in wine samples was also analyzed by SensLab-biosensor [27] and FC *b*_2_-based method [20, 21]. The obtained analytical results were compared with L-lactate content declared by a producer. The wine samples *Cabernet Sauvignon*, *Chardonnay,* and *Sherry Strong* received from *Magarach* winery (the Crimea, Ukraine) were used for such experiments (Table 3).

The significant difference between L-lactate content in the wine *Chardonnay* declared by the producer and obtained by biosensor and current FC *b*_2_-based method could be explained by a low selectivity of the routine used in winery (low-resolution ion-exchange chromatography coupled with colorimetric) analysis.

## 4. Conclusions

A new enzymatic method for L-lactate analysis has been proposed. The method is based on enzymatic oxidation of L-lactate to pyruvate in the presence of flavocytochrome *b*_2_ coupled with nitrotetrazolium blue reduction to colored product, formazan. Optimal conditions for carrying out correct L-lactate analysis have been found. The main advantages of the proposed method when compared to the LDH-based routine approaches are a higher sensitivity (2.0 *μ*M of L-lactate), simple procedure of analysis, and usage of inexpensive, nontoxic reagents and small amount of the enzyme. The method has been used for quantitative determination of L-lactate in food products and wine samples. A good correlation for L-lactate analysis between results obtained by current FC *b*_2_-based method and other selective analytical methods clearly shows a practical importance of the developed method for biotechnology and food technology.

## Figures and Tables

**Table 1:** Bioanalytical characteristics of different methods of L-lactate assay (calculated for concentrations in the final reaction mixture).

Enzymatic methods	*λ*_max⁡_, nm	*E*, mM^−1^ cm^−1^	Detection limit, *μ*M	Linear range, mM	Time of analysis, min
LDH-GPT based [24]	340	6.3	3.37	0.033–0.39	30
Flavocytochrome *b*_2_-based [20]	680	5.0	3.0	0.008–0.27	30
LO: peroxidase; ABTS based [25]	405	34	—	0.002–0.068	30
LDH based [14]	340	6.3	5.6	0.025–0.16	30
Lactate oxidase and aminoantipyrine based [26]	546	38	—	0.0003–0.099	10
Current method	525	12	2.0	0.005–0.14	20

**Table 2:** Comparison of the results of L-lactate assay (in mM) in food products.

Sample	Method			
	*SensLab* biosensor [27]	FC *b*_2_-based method [20, 21]	NAD^+^-LDH-based method [28]	Current method
*Rewe* Milk (0.3% fat)	0.21 ± 0.02 *P* > 0.05**	0.20 ± 0.05 *P* > 0.05**	0.278 ± 0.03 *P* < 0.05*	0.192 ± 0.002
*Milsani* Milk (1.5% fat)	0.16 ± 0.04 *P* > 0.05**	0.179 ± 0.006 *P* > 0.05**	0.26 ± 0.33 *P* < 0.05*	0.172 ± 0.01
*Rich* Ketchup	1.43 ± 0.35 *P* > 0.05**	1.36 ± 0.02 *P* > 0.05**	1.64 ± 0.11 *P* > 0.05**	1.46 ± 0.4
*Maitre* Ketchup	0.62 ± 0.129 *P* > 0.05**	0.78 ± 0.01 *P* > 0.05**	1.08 ± 0.04 *P* > 0.05**	0.74 ± 0.02
*Krings* Apple juice 100%	0.15 ± 0.014 *P* > 0.05**	0.14 ± 0.04 *P* > 0.05**	0.256 ± 0.05 *P* < 0.05*	0.16 ± 0.015
*Belsina* Apple drink (50%)	0.14 ± 0.001 *P* > 0.05**	0.132 ± 0.01 *P* > 0.05**	0.285 ± 0.07 *P* < 0.05*	0.135 ± 0.01

*Difference between current method and the compared methods is statistically significant.

**Difference between current method and other methods is statistically insignificant.

**Table 3:** Comparison of the results of L-lactate assay (in g·L^−1^) in wine samples.

Sample	Method			
	*SensLab *biosensor [27]	FC *b*_2_-based method [20, 21]	Declared by producer	Current method
*Cabernet Sauvignon* (dry red)	2.4 ± 0.28 *P* > 0.05**	2.25 ± 0.18 *P* > 0.05**	2.5 ± 0.2 *P* > 0.05**	2.15 ± 0.13
*Chardonnay* (dry white)	1.16 ± 0.11 *P* > 0.05**	1.03 ± 0.08 *P* > 0.05**	3.0 ± 0.2 *P* < 0.05*	0.97 ± 0.12
*Sherry* (strong white)	0.58 ± 0.05 *P* > 0.05**	0.6 ± 0.07 *P* > 0.05**	1.1 ± 0.2 *P* < 0.05*	0.49 ± 0.08

*Difference between current method and the compared methods is statistically significant.

**Difference between current method and other methods is statistically insignificant.
